# Assessment of the relationship between diabetes treatment intensification and quality measure performance using electronic medical records

**DOI:** 10.1371/journal.pone.0199011

**Published:** 2018-06-12

**Authors:** Renée J. G. Arnold, Shuo Yang, Edward J. Gold, Sepehr Farahbakhshian, John J. Sheehan

**Affiliations:** 1 Quorum Consulting, Inc., New York, New York, United States of America; 2 Icahn School of Medicine at Mount Sinai, New York City, New York, United States of America; 3 Old Hook Medical Associates, Emerson, New Jersey, United States of America; 4 AstraZeneca, Wilmington, Delaware, United States of America; University of Tennessee Health Science Center, UNITED STATES

## Abstract

**Aims:**

Assess the relationship between timely treatment intensification and hemoglobin A1C (HbA1C) control quality-of-care performance measures, i.e., HbA1C levels, among patients with uncontrolled type 2 diabetes.

**Materials and methods:**

Electronic medical records and diabetes registry data from a large, accountable care organization (ACO) were used to isolate a sample of adult patients with type 2 diabetes who received at least one oral antidiabetes agent and had at least one HbA1C level measurement ≥8.0% (64 mmol/mol; i.e., uncontrolled diabetes) between 7/1/2011 and 6/30/2015. Treatment intensification status was evaluated for each patient during a 120-day treatment intensification window following the index HbA1c measure. Two-level hierarchical generalized linear models, with patients aggregated at the physician level, were used to assess the association between treatment intensification and achieving HbA1C quality performance measures.

**Results:**

547 patients met study selection criteria and 480 patients had at least one HbA1C test after the treatment intensification window and were used for the statistical analyses. About 40% of patients who had uncontrolled diabetes received treatment intensification during the 120-day window. Greater index HbA1C, greater patient body mass index, and fewer unique pre-index oral antidiabetes agents were significantly associated with greater likelihood of receiving timely treatment intensification. The odds of receiving treatment intensification were about 1.8 times higher (P = 0.0027) among patients with poor index HbA1C control (HbA1c level >9.0% [75 mmol/mol]) compared to other patients (index HbA1c 8.0% - 9.0%). Hispanic patients (compared to White patients) were significantly more likely to exhibit poor control after treatment intensification (odds ratio [OR] 2.91, P = 0.0304), underscoring the difficulty of controlling diabetes in this vulnerable group. In contrast, being male and being treated primarily by an internist (compared to primary treatment by a family medicine specialist) were both significantly associated with achieving superior control (HbA1c level <8.0%) after treatment intensification (OR 0.53 [P = 0.0165]; OR 0.41 [P = 0.0275], respectively).

**Conclusions:**

Timely treatment intensification was significantly associated with greater likelihood of patients achieving superior HbA1C control (<8.0%) and better HbA1C control quality performance for the practice. Even in an ACO with resources dedicated to diabetes control, it is incumbent upon clinicians to readily identify and open dialogues with patients who may benefit from closely supervised, individualized attention.

## Introduction

The increasing prevalence of type 2 diabetes, and the large health and economic burden of this disease on both patients and society, make type 2 diabetes a pressing public health issue in the United States (US). Quality assessment measures such as those developed by the National Committee for Quality Assurance (NCQA) have been used to evaluate healthcare delivery and management for providers and health plans to guide patient care and address public health issues on a health system level. Even among therapeutic areas classified as “high-impact” based on prevalence, mortality, and costs by the National Pharmaceutical Council, the largest number of direct and indirect NCQA quality measures pertain to diabetes, demonstrating the continued need and national effort to improve diabetes care and management in the US [1].

Hemoglobin A1C (HbA1C) control is an essential part of care for type 2 diabetes and, among other quality measures, is a key component of all quality measure performance assessment. The Healthcare Effectiveness Data and Information Set (HEDIS) defines poor diabetes control as HbA1C >9.0% (75 mmol/mol) and good diabetes control as HbA1C <8.0% (64 mmol/mol) in adults 18–75 years of age. The Physician Quality Reporting System (PQRS) encourages reporting the percentage of diabetes patients with poor control [2].

The value of timely treatment intensification for diabetes patients with above target HbA1C levels has been well-documented. Several studies showed that patients who received more intensive therapy or had their medications adjusted in a timely fashion improved their HbA1C levels [3–6]. The treatment guidelines from the American Diabetes Association (ADA) and American Association of Clinical Endocrinologists (AACE) recommend treatment intensification for patients not at treatment goal after three months of therapy [7, 8]. Even with abundant evidence of the value of treatment adjustment, failure of treatment intensification among patients with above-target HbA1C is commonly observed. One US database study found that median time to treatment intensification was almost two years [9]. An international study with longer follow-up found time to treatment intensification could be greater than seven years [10].

Several factors are associated with diabetes treatment inertia, which is defined as failure to intensify pharmacotherapy in accordance with clinical guidelines. Recent studies using primary care populations suggest that complexity of treatment [11], patient medication adherence [12], HbA1C level [13], specialty of the physician initiating the therapy [14], physician experience level [13], and other provider-, patient- and system-level barriers [15] may be linked to diabetes treatment inertia. While no study has assessed the direct relationship between timely treatment intensification and diabetes quality measure performance, it is reasonable to expect that timely treatment intensification would contribute to greater likelihood of achievement of HbA1C quality performance goals as measured by HbA1C control.

Given the treatment inertia observed in the above studies, increased adherence to treatment guidelines and timely treatment intensification for patients with uncontrolled type 2 diabetes may greatly improve quality measure performance for health plans and providers. Compared to population-level databases, electronic medical record (EMR) data for a registry population from a physician practice would be expected to have more complete data, including labs and vital data, to support an evaluation of this relationship, thereby enhancing both validity and applicability of the results to clinical practice management. In addition, since the EMR is used for daily patient encounters, the data should be of higher quality, although there may still be missing data. This study aimed to assess the relationship between diabetes treatment intensification and HbA1C control quality-of-care measures among patients with uncontrolled type 2 diabetes.

## Materials and methods

### Study population

**Fig 1:**
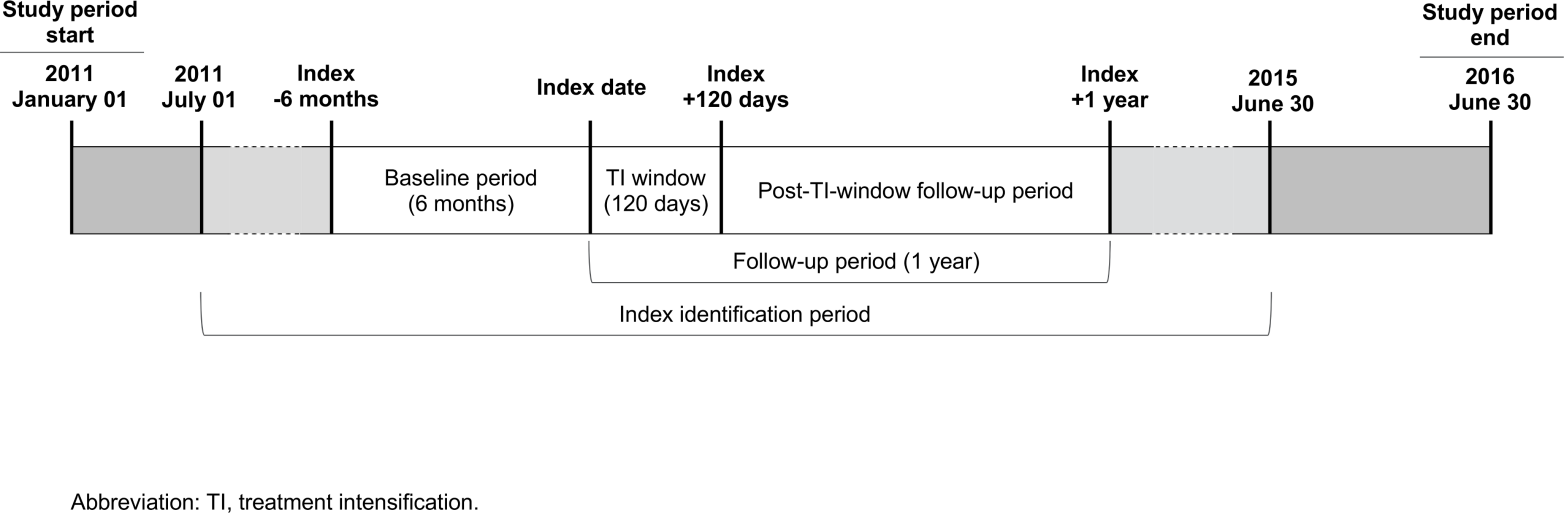
Study design and timeframe.

This retrospective study used de-identified EMR and diabetes registry data from an ACO located in Bergen County, New Jersey, USA. The patient population served by this practice are fully-insured and primarily are in the middle to upper-middle socioeconomic class. The study period was from January 1, 2011 to June 30, 2016, with a patient index identification period from July 1, 2011 to June 30, 2015. Index date for each included patient was defined as the date of the first above-target HbA1C test (i.e., ≥8.0%) within the index identification period. The baseline period was defined as the six months prior to index and the follow-up period was one year following index (Fig 1). Within the follow-up period, the treatment intensification window was defined as the first 120 days following study index date.

**Fig 2:**
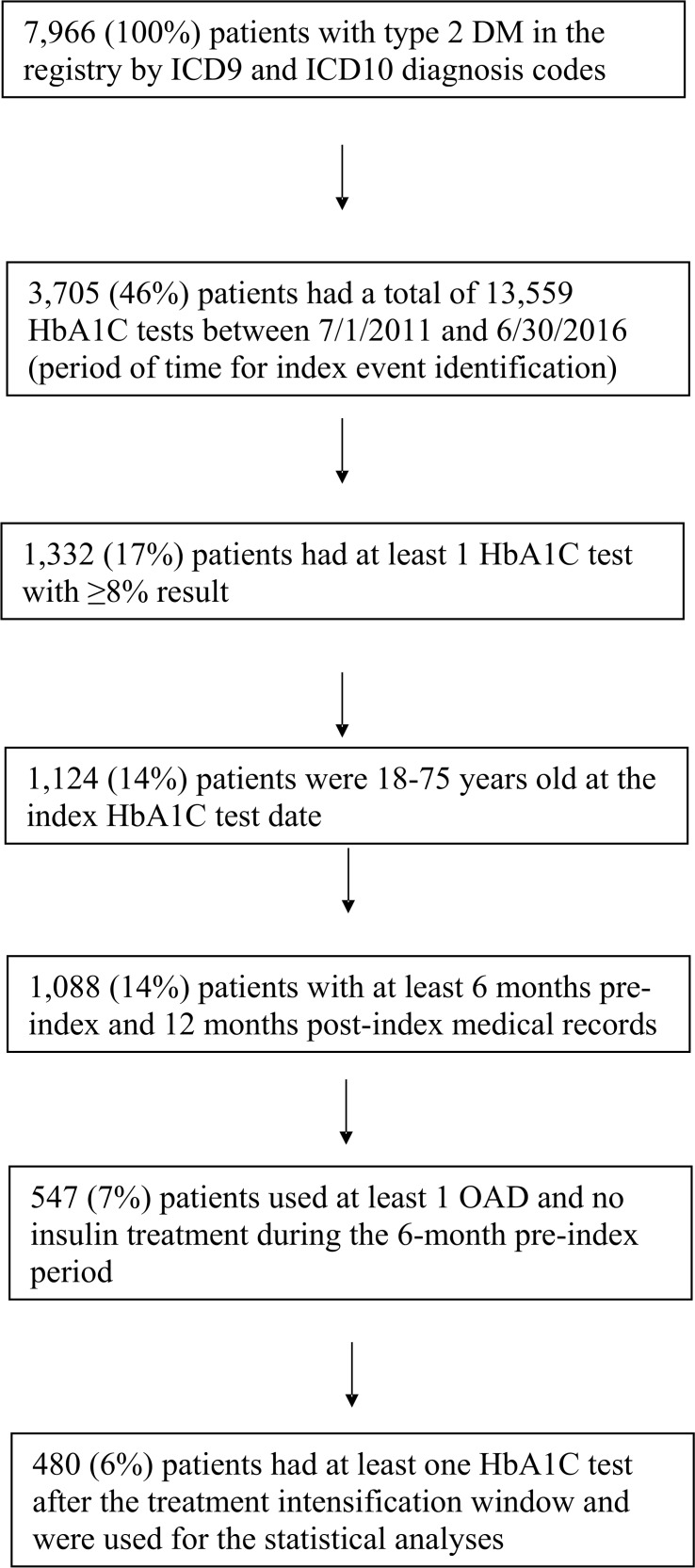
Selection flow diagram demonstrating identification and derivation of the final patient cohorts.

Included patients were required to a) have physician-diagnosed type 2 diabetes; b) have had an HbA1C level ≥8.0%, which was considered uncontrolled diabetes, during the study period; c) be 18–75 years of age at index date; d) have had at least 6 months of medical records prior to index date (baseline period) and 1 year of medical records after index date (follow-up period); and e) have used at least one oral antidiabetes (OAD) agent during the 6-month baseline period (Fig 2). Patients who were on insulin treatment during the 6-month baseline period were excluded from the study (Fig 2). OAD drug classes examined included biguanides, sulfonylureas, dipeptidyl peptidase-4 (DPP-4) inhibitors, insulin-sensitizing agents, sodium glucose cotransporter-2 (SGLT2) inhibitors, meglitinides, bile acid sequestrants, and alpha glucosidase inhibitors, alone or in combination. As all data were deidentified, institutional review board (IRB) approval was not needed.

### Outcome measures

The presence or absence of timely adjustments to treatment for each uncontrolled diabetes patient was assessed based on the patient’s treatment intensification status and practice quality performance status was assessed based on patients’ HbA1c control categories during the follow-up period. Patients’ diabetes treatment intensification statuses were evaluated using the post-index medication history for each included patient. Following the above target index HbA1C level (≥8.0%), treatment intensification was defined as at least one medication treatment change that satisfied one or more of the following conditions: 1) adding one or more OADs to the existing treatment; 2) adding or switching to an injectable antidiabetes medication; or 3) increasing the dosage of existing OADs. Lack of treatment intensification within the 120-day window after index date was considered treatment inertia. In the case of multiple medication changes qualifying as treatment intensification, the date of the earliest change was documented as the date of treatment intensification. Physician/patient characteristics and their associations with treatment intensification status were evaluated among all patients included in the study population (N = 547). Patients chose their primary care physician, who then determined if the patient needed to be seen by an endocrinologist.

To determine the quality performance of the medical practice, the degree of HbA1c control in the follow-up period was determined based on the results of the first HbA1C test conducted after the end of the treatment intensification window and more than 30 days following the treatment intensification date, if applicable [16]. Patients with HbA1C levels <8.0%, between 8.0% and 9.0%, or >9.0%) were defined as the cohort with superior, moderate, or poor HbA1C control, respectively. In addition, models testing the association between treatment intensification status, physician/patient attributes and HbA1c control category were evaluated among the subset of the study population that had at least one qualifying HbA1C test during the follow-up period after the treatment intensification window (N = 480).

### Sensitivity analysis

At the end of each calendar year, patients’ most recent HbA1C levels are used in annual quality-of-care performance reports to determine the quality performance of the medical practice. Thus, patients’ HbA1C control categories—poor, moderate or superior—were also examined at year-end in a sensitivity analysis using the last (as opposed to the first) HbA1c level measured during each patient’s follow-up period after the treatment intensification window.

### Statistical analysis

Patient baseline demographics and clinical characteristics–including age, gender, race/ethnicity, insurance type, body mass index (BMI), comorbid conditions other than diabetes, and the number and dose of OADs used at baseline–were extracted from the EMR system and registry data. Descriptive analyses were conducted on the baseline patient characteristics by treatment intensification status or HbA1C control category. Analyses of performance quality were conducted using categories of HbA1c control based on the first HbA1c test result during the follow-up period after the treatment intensification window (base case analysis) and using categories based on the last HbA1c test result (sensitivity analyses).

In addition to the descriptive analyses, a two-level hierarchical generalized linear model was used to examine the relationship between patients’ baseline characteristics, physicians’ characteristics (such as age, gender, specialty, number of years in practice, and average patient volume per month), and patients’ treatment intensification status. Two-level hierarchical generalized linear models were also used to examine the association between patients’ treatment intensification status and HbA1C control performance measures (HbA1C control categories), with the immediate next level after the treatment intensification window. Comparisons between groups were made using Wilcoxon rank-sum tests for continuous variables and chi-squared tests (or Fisher’s exact tests) for categorical variables, as appropriate. Confidence intervals were calculated for all appropriate variables. All statistical analyses were performed using SAS 9.4 (SAS Institute, Inc., Cary, NC, USA).

## Results

A total of 547 patients with type 2 diabetes were identified after applying all inclusion and exclusion criteria; of those, 480 patients had at least one HbA1C test after the treatment intensification window and were included for statistical analyses that incorporated an HbA1C test result (Fig 2). Evaluations of patient baseline characteristics by treatment intensification status (Table 1) demonstrated that a greater proportion of patients in the cohort with poor HbA1C control at the index test received treatment intensification than patients in the cohort with superior HbA1C control (P = 0.0016). BMI was higher among patients who received treatment intensification compared to patients who did not (P = 0.0139). Despite having above-target HbA1c levels at index, only 39.9% of patients received treatment intensification during the 120-day window.

**Table 1:** Patient and provider baseline characteristics.

	All Patients (N = 547)	Treatment Intensification		
		No (N = 329)	Yes (N = 218)	P-value
**Index HbA1C result category**				0.0016*
Moderate HbA1C control	311	205 (65.92%)	106 (34.08%)	
Poor HbA1C control	236	124 (52.54%)	112 (47.46%)	
**Age (years)**				0.3688
Mean (SD)	58.56 (9.74)	58.40 (9.41)	58.82 (10.23)	
**Sex**				0.986
Male	344	207 (60.17%)	137 (39.83%)	
**Race/Ethnicity**				0.4716
White	372	217 (58.33%)	155 (41.67%)	
Hispanic	43	26 (60.47%)	17 (39.53%)	
Black	38	27 (71.05%)	11 (28.95%)	
Asian	24	17 (70.83%)	7 (29.17%)	
Other/Unknown	70	42 (60.00%)	28 (40.00%)	
**CCI**				0.2243
Mean (SD)	1.39 (0.92)	1.35 (0.89)	1.46 (0.96)	
**CCI category**				0.0909
1	433	265 (61.20%)	168 (38.8%)	
2	48	32 (66.67%)	16 (33.33%)	
3+	66	32 (48.48%)	34 (51.52%)	
**BMI**				0.0139*
Mean (SD)	32.98 (6.76)	32.40 (6.63)	33.85 (6.87)	
**Insurance type**				0.3137
Commercial	375	229 (61.07%)	146 (38.93%)	
Medicare	169	97 (57.40%)	72 (42.60%)	
Other/Unknown	3	3 (100%)		
**Patient assigned provider specialty**				0.4448
Endocrinology, Diabetes & Metabolism	215	131 (60.93%)	84 (39.07%)	
Internal Medicine	160	102 (63.75%)	58 (36.25%)	
Family Practice	111	60 (54.05%)	51 (45.95%)	
All other specialties	61	36 (59.02%)	25 (40.98%)	
**Number of OAD types used at baseline**				0.0027*
1	248	131 (52.82%)	117 (47.18%)	
2	196	123 (62.76%)	73 (37.24%)	
3	80	56 (70.00%)	24 (30.00%)	
4	23	19 (82.61%)	4 (17.39%)	
**All physicians characteristics by gender**				
Provider Gender	Total	Male (%)	Female (%)	P-value
	58	35 (60.34)	23 (39.66)	0.1151
Provider Age (years)				
Mean (SD)	53.4 (17.00)	59.5 (17.35)	44.2 (11.62)	0.0005*
Provider Specialty				0.2491
Internal Medicine	29 (50)	17 (48.57)	12 (52.17)	
Family practice	11 (18.97)	8 (22.86)	3 (13.04)	
Endocrinology	7 (12.07)	3 (8.57)	4 (17.39)	
Gastroenterology	4 (6.9)	4 (11.43)	0 (0)	
Dermatology	2 (3.45)	0 (0)	2 (8.7)	
Podiatry	2 (3.45)	1 (2.86)	1 (4.35)	
Cardiology	1 (1.72)	1 (2.86)	0 (0)	
GYN	1 (1.72)	0 (0)	1 (4.35)	
Otolaryngology	1 (1.72)	1 (2.86)	0 (0)	

* P<0.05

Abbreviations: BMI- body mass index; CCI- Charlson Comorbidity Index; HbA1C- hemoglobin A1C; OAD- oral antidiabetes agent; SD- standard deviation

After the treatment intensification window, patients’ HbA1C control status was examined by comparing the cohorts with superior and poor control. The odds of receiving treatment intensification were about 1.8 times higher (Table 2) among the cohort with poor HbA1C control in comparison to the cohort with moderate HbA1C control, P = 0.0027. The more types of OADs used at baseline, the less likely patients received treatment intensification, with P values of 0.0058, 0.0102 and 0.0119 for 4, 3 and 2 OAD types, respectively. Patients with higher BMI were more likely to receive treatment intensification (P = 0.0159). No other physician or patient factors were significantly associated with treatment intensification. After accounting for the difference in patient factors, the probability of treatment intensification varied by physician, but the result was not statistically significant. Timely treatment intensification was significantly associated with superior HbA1C control (Table 3) but not with poor HbA1C control (Table 4). Hispanic patients were approximately three-fold more likely than White patients to still have poor HbA1C control after treatment intensification, and male patients were approximately half as likely as female patients to experience poor HbA1C control after treatment intensification (Table 4).

**Table 2:** Physician and patient characteristics associated with type 2 diabetes treatment intensification.

	Odds Ratio (95% Confidence Interval)	P-value
**Physician characteristics **		
**Age**	0.9362 (0.8761–1.0004)	0.052
**Male (compared to female)**	1.2268 (0.6957–2.1633)	0.4803
**Specialty (compared to Family Medicine)**		
Endocrinology, Diabetes & Metabolism	1.1138 (0.6493–1.9105)	0.6957
Internal Medicine	0.8239 (0.4666–1.4548)	0.5047
All other specialties	0.9049 (0.445–1.84)	0.7826
**Year in practice**	1.0626 (0.9937–1.1362)	0.0764
**Average patient volume per month**	1.0013 (0.9989–1.0037)	0.2888
**Patient characteristics**		
**Age**	1.0123 (0.9877–1.0374)	0.3318
**Male (compared to female)**	0.9159 (0.6145–1.3651)	0.6663
**Race/Ethnicity (compared to White)**		
Black	0.6556 (0.2932–1.4658)	0.3043
Hispanic	1.4283 (0.6557–3.1112)	0.3699
All others	0.9226 (0.5618–1.5152)	0.7505
**BMI**	1.0344 (1.0064–1.0631)	0.0159*
**CCI category (compared to 1)**		
2	0.6372 (0.3255–1.2477)	0.1893
3+	1.4592 (0.8291–2.5681)	0.1906
**Insurance type (compared to Commercial)**		
Medicare & Others	1.056 (0.6476–1.7221)	0.8271
**Index HbA1C results category (compared to moderate HbA1C control)**		
Poor HbA1C control	1.7834 (1.2253–2.5957)	0.0027*
**Number of OAD types used at baseline (compared to 1)**		
2	0.5874 (0.3887–0.8877)	0.0119*
3	0.4712 (0.266–0.8346)	0.0102*
4	0.1928 (0.0601–0.6181)	0.0058*

* P<0.05

Abbreviations: BMI- body mass index; CCI- Charlson Comorbidity Index; HbA1C- hemoglobin A1C; OAD- oral antidiabetes agent

**Table 3:** Association of treatment intensification with superior HbA1C control, using the immediate next level after treatment intensification window (N = 480).

	Odds Ratio (95% Confidence Interval)	P-value
Timely treatment intensification	1.8107 (2.7661–1.8107)	0.0063*
**Physician characteristics**		
**Age**	0.9887 (1.0612–0.9887)	0.7536
**Male (compared to female)**	0.5738 (1.0899–0.5738)	0.0905
**Physician specialty (compared to Family Medicine)**		
Endocrinology, Diabetes & Metabolism	1.1626 (2.1142–1.1626)	0.6217
Internal Medicine	1.6021 (2.9967–1.6021)	0.1409
All other specialties	1.8426 (3.9559–1.8426)	0.1177
**Year in practice**	1.026 (1.1027–1.026)	0.4855
**Average patient volume per month**	1.0011 (1.0039–1.0011)	0.4215
**Patient characteristics**		
**Age**	1.0146 (1.0431–1.0146)	0.3073
**Male (compared to female)**	1.3875 (2.162–1.3875)	0.1486
**Race/Ethnicity (compared to White)**		
Black	1.3222 (3.093–1.3222)	0.5199
Hispanic	0.5045 (1.2381–0.5045)	0.136
All others	1.7333 (2.9841–1.7333)	0.0479*
**BMI**	0.9729 (1.0037–0.9729)	0.0848
**CCI category (compared to 1)**		
2	0.7947 (1.5989–0.7947)	0.5197
3+	1.029 (1.9459–1.029)	0.9301
**Insurance type (compared to Commercial)**		
Medicare & Other	1.0001 (1.6971–1.0001)	0.9996
**Index HbA1C results category (compared to moderate HbA1C control)**		
>9.0% (75 mmol/mol)	0.2452 (0.3758–0.2452)	< .0001*
**Number of OAD types used at baseline (compared to 1)**		
2	0.6061 (0.9605–0.6061)	0.0336*
3	0.6501 (1.1801–0.6501)	0.1577
4	0.9932 (2.8359–0.9932)	0.9898

* P<0.05

Abbreviations: BMI- body mass index; CCI- Charlson Comorbidity Index; HbA1C- hemoglobin A1C; OAD- oral antidiabetes agent

**Table 4:** Association of treatment intensification with poor HbA1C control, using the immediate next level after treatment intensification window (N = 480).

	Odds Ratio (95% Confidence Interval)	P-value
Timely treatment intensification	0.741 (0.4576–1.1999)	0.2236
**Physician characteristics**		
**Age**	1.0026 (0.9171–1.096)	0.9545
**Male (compared to female)**	1.5299 (0.6773–3.4555)	0.307
**Physician specialty (compared to Family Medicine)**		
Endocrinology, Diabetes & Metabolism	0.6523 (0.3014–1.412)	0.2788
Internal Medicine	0.4125 (0.1882–0.9039)	0.0275*
All other specialties	0.4893 (0.1858–1.2891)	0.1489
**Year in practice**	0.9817 (0.8968–1.0746)	0.6892
**Average patient volume per month**	0.9967 (0.9932–1.0002)	0.0657
**Patient characteristics**		
**Age**	0.9924 (0.962–1.0237)	0.6288
**Male (compared to female)**	0.5345 (0.3208–0.8904)	0.0165*
**Race/Ethnicity (compared to White)**		
Black	0.6819 (0.2447–1.9008)	0.4646
Hispanic	2.9066 (1.1097–7.6137)	0.0304*
All others	0.6754 (0.3576–1.2755)	0.227
**BMI**	1.0226 (0.988–1.0584)	0.2041
**CCI category (compared to 1)**		
2	1.4049 (0.643–3.0699)	0.3944
3+	1.2496 (0.5979–2.6116)	0.5539
**Insurance type (compared to Commercial)**		
Medicare & Other	0.7584 (0.4067–1.4145)	0.3851
**Index HbA1C results category (compared to moderate HbA1C control**		
Poor HbA1C control	5.4734 (3.3809–8.8611)	< .0001*
**Number of OAD types used at baseline (compared to 1)**		
2	1.3218 (0.7793–2.2421)	0.3013
3	1.3197 (0.6628–2.6278)	0.4304
4	0.7453 (0.2058–2.6986)	0.6545

* P<0.05

Abbreviations: BMI- body mass index; CCI- Charlson Comorbidity Index; HbA1C- hemoglobin A1C; OAD- oral antidiabetes agent

Compared with the family medicine physician specialty, internal medicine physician specialty was associated with a lower likelihood of patients having poor HbA1C control after the treatment intensification window (P = 0.0275) (Table 4). A higher index HbA1C level was consistently associated with worse HbA1C control status after the treatment intensification window in all four models. After accounting for differences in patient characteristics, variability between different physician specialties was not significant in any of the four models.

Patients who received treatment intensification had a higher average number of HbA1C tests during their follow-up period compared with patients who did not (mean [SD]: 2.49 [0.89] vs. 2.26 [0.9]; P = 0.0036).

### Sensitivity analysis

HbA1c control after the treatment intensification window was better when assessed based on the last HbA1c level tested during the patients’ one-year follow-up period (i.e., the value that would contribute to a year-end annual report), than when assessed using the first HbA1c level tested following the end of the patients’ treatment intensification windows (S1 and S2 Tables for the cohort with superior HbA1C control and S3 and S4 Tables for the cohort with poor HbA1C control). For the four different measures, it was consistent that the cohort with moderate HbA1C control was more likely to have better HbA1C control at follow up; patients with better HbA1C control also appeared to be older (S1–S4 Tables). As with the base case analysis using the first HbA1C test after the treatment intensification window, timely treatment intensification was also associated with superior HbA1C control (S5 Table) but not with poor HbA1C control (S6 Table) at the last HbA1c level tested during the patients’ annual follow-up periods.

## Discussion

After an above-target HbA1C level (≥8.0%), more than 60% of patients with type 2 diabetes still did not receive treatment intensification (Table 1). Patients with higher index HbA1C levels, however, were more likely to receive timely treatment intensification, a result supported by the findings in similar studies [17, 18]. Unlike another study, which showed that younger patients were more likely to undergo treatment intensification, the current study (Table 2) showed that patient age had no bearing on the likelihood of the patient regimen being intensified [17]. In addition, in the current study, patients receiving fewer unique OAD agents were more likely to receive timely treatment intensification (Table 2). These findings fall towards the better end of the range of previously published literature [11]. Previous large retrospective studies suggest that anywhere between 56% and 73% of patients with type 2 diabetes do not receive treatment intensification within 3 to 6 months following the index event [6, 9, 14, 17]. This is similar to the current study, where more than 60% of patients did not receive treatment intensification within 4 months after an above-target HbA1C level ≥8.0%. As shown in the current study and in other previous studies, patients who receive treatment intensification within 6 months have significantly greater HbA1C reduction (-0.33%) within 1 year of follow-up compared to patients who do not receive treatment intensification [6].

A previous study conducted in France by Balkau et al. used electronic records (2008–2009) of a panel of French general practitioners [17]. Similar to the current study, they conducted univariate and multivariate statistical analyses using Cox proportional hazard models to identify associations between patient or practitioner characteristics and treatment intensification. In their study, factors associated with treatment intensification included age 65–79 years (hazard ratio [HR]: 0.71, P<0.001), and age ≥ 80 years (HR: 0.50, P: <0.001), but not age <65 years; treatment with bitherapy (HR: 1.19, P = 0.005) but not tritherapy, all in comparison to monotherapy; the first HbA1c above the recommended target (7.01–8.5 HR: 1.51 [P <0.001]; 8.51–9.5 HR: 2.16 [P <0.001]; ≥9.51 HR: 1.38 [P = 0.04]), and no treatment for hypertension (HR for patients with treatment: 0.82, P = 0.003).

BMI was not examined in the previous study by Balkau et al., but the current study found that BMI, index HbA1C control category and number of OAD types used at baseline were associated with treatment intensification (Table 2). Patients with higher BMI were more likely to receive treatment intensification (P = 0.0159). The odds of receiving treatment intensification were about 1.8 times higher (Table 2) in the cohort with poor HbA1C control than in the cohort with moderate HbA1C control(P = 0.0027). The more types of OADs used at baseline, the less likely it was for patients to receive treatment intensification (Table 2). Our study found that no other physician or patient factors were associated with treatment intensification, similar to the findings of Balkau and colleagues [17].

Differences between the current and the previous study may have been due to country, data used, patient selection criteria and categorization of HbA1C levels. While our study used 8.0%-9.0% to indicate moderate HbA1C control and >9.0% to indicate poor HbA1C control, Balkau et al. utilized categories of ≤ 7.0, 7.01–8.5, 8.51–9.5, ≥9.51.

Most previous studies conducted to identify predictors of treatment intensification used data sources like EMR [17, 19], administrative claims [9], diabetes treatment database [20], pharmacy dispensing data [21] or a combination of survey, EMR and claims data [22]. One study conducted a prospective analysis of patients enrolled in a managed care program [23]. Major differences between the previous studies included baseline HbA1C levels of the included patients, OAD or insulin use at baseline [9, 17], inclusion/exclusion criteria, the gap between HbA1C tests [17], choice of variables considered and type of statistical analyses conducted. These studies revealed that treatment intensification was associated with the number of OAD types used at baseline [17, 23], younger age [17, 19, 22], higher HbA1C values at baseline [9, 17, 19, 22], longer duration of diabetes [19, 20, 22], point-of-service insurance, mental illness, endocrinologist visit at baseline [9], no treatment for hypertension [17], using insulin at baseline [19], non-adherence to OADs [21], worse glycemia, and patients with longer intervals between routine hyperglycemic visits [17, 23]. Most studies indicated no association between physician-related factors and treatment intensification [17, 19]. Studies also concluded that higher baseline HbA1C, older age, black ethnicity, lower income, and more physician visits were associated with improvement in HbA1C levels after treatment intensification [22]. As indicated in other studies, the current study also showed that both ethnicity (Hispanic) and being female were significantly associated with poor HbA1c control after treatment intensification (Table 4).

In addition, after controlling for available patient and physician factors, no significant variability on the physician level was found in our study, except that patients followed by internists were about half as likely as those followed by family medicine specialists to exhibit poor HbA1c control (Table 4). This is similar to other published literature, which showed no difference in HbA1C control between patients treated by family medicine specialists compared to endocrinologists, [24]; this finding may be attributed to the fact that the physicians examined in the study were from the same practice facility. Furthermore, the fact that this practice attempts to follow the recommended treatment guidelines may have reduced the variability in process and outcomes of patient care. Nonetheless, the study did confirm that timely treatment intensification was significantly associated with superior patient HbA1C control during follow-up. Although an association was not observed for the cohort with poor HbA1C control, this is very likely due to the fact that the study used “patient had an HbA1C level ≥8.0%” as the criterion to trigger treatment intensification instead of using >9.0% levels.

In response to the current study’s findings, the practice has instituted a program using the health maintenance section and practice analytics module of their EMR. They have programmed the system to flag patients with elevated HbA1C levels and send out reminder letters. They also have a “care coordinator” directly contact those patients with elevated HbA1C levels who have not been seen or do not have a scheduled appointment within 3 months to schedule follow-up.

There were several limitations to the study, beginning with the data being from a regional ACO. Although the patient population was diverse in its demographics, care must be taken in generalizing the results. In addition, the study used HbA1C levels ≥8.0% as the selection criterion for the base patient population. This is consistent with the practice’s internal recommendations for treating patients with type 2 diabetes and the outcome measure of HbA1C control status; however, it may not necessarily be closely related to the HbA1C poor control performance measure. ADA and AACE guidelines both recommend treatment intensification for patients not at treatment goal after 3 months of therapy [7, 8]. Considering the treatment patterns in real-world practice, the study used a 120-day treatment intensification window rather than a 90-day window. In addition, it was possible that patients may not have filled the prescription prescribed as part of the treatment intensification regimen and, in that case, treatment inertia could be underestimated. Unfortunately, the EMR data did not include patient fill, thereby preventing determination of treatment adherence. At the same time, treatment intensification that was missing from the EMR data could be categorized as treatment inertia and cause overestimation of treatment inertia. Even so, the data did provide a very valuable perspective that enabled this evaluation of clinicians’ prescribing practices and HbA1C control. Regardless of fill status, these results support an association between the two and add credibility to the value of outcome-based quality measures. Lastly, it would be very hard to experimentally determine if clinical contact causatively results in better HbA1C outcomes, as only motivated patients may consent. The results here suggest the value of regular clinical contact and monitoring, which was associated with better HbA1C control.

This research is the first published instance of primary care EMR data used to evaluate treatment intensification and its effect on diabetes control and adherence to quality measures, specifically HbA1C. The study shows that it is possible to collect and analyze these data to determine how to positively influence physician behavior to improve patient outcomes. Unlike previous studies, one of the key advantages of the current study is the fact that the data set was very complete. The Chief Medical Officer of the practice has been a proponent of EMR use for the past 20 years and all prescriptions that go out must go through the EMR. Given that this retrospective study evaluated a relatively affluent suburban population and that there were 7 endocrinologists on staff at the practice during the study period, it is daunting that the treatment inertia was as high as it was—underscoring the intractability of timely treatment for patients with type 2 diabetes. There are likely psychosocial and other unmeasured patient and physician factors at play here, which should be more closely examined in future studies.

Although researchers typically rely on large datasets available commercially, the current research showed results consistent with previous publications using a dataset available to practicing clinicians on a daily basis in their practices. The use of a practice’s patient data for important research such as this demonstrates that these types of analyses are not restricted to researchers with large data sets, but can also be used to evaluate outcomes for individual clinicians and practices.

## Conclusions

Timely treatment intensification was significantly associated with greater likelihood of patients achieving superior HbA1C control (<8.0%) and better HbA1C control quality performance for the practice. Even in a large group practice with resources dedicated to diabetes control, it is incumbent upon clinicians to readily identify and open dialogues with patients who may benefit from closely supervised, individualized attention.

## Supporting information

S1 Table Patients’ treatment intensification and baseline characteristics by superior HbA1C control status, using the immediate next level after treatment intensification window. Abbreviations: BMI- body mass index; CCI- Charlson Comorbidity Index; OAD- oral antidiabetes agent; SD- standard deviation.
(DOCX)

S2 Table Patients’ treatment intensification and baseline characteristics by superior HbA1C control status, with the level used in the next annual performance report. Abbreviations: BMI- body mass index; CCI- Charlson Comorbidity Index; OAD- oral antidiabetes agent; SD- standard deviation.
(DOCX)

S3 Table Patients’ treatment intensification and baseline characteristics by poor HbA1C control status, using the immediate next level after treatment intensification window. Abbreviations: BMI- body mass index; CCI- Charlson Comorbidity Index; OAD- oral antidiabetes agent; SD- standard deviation.
(DOCX)

S4 Table Patients’ treatment intensification and baseline characteristics by poor HbA1C control status, with the level used in the next annual performance report. Abbreviations: BMI- body mass index; CCI- Charlson Comorbidity Index; OAD- oral antidiabetes agent; SD- standard deviation.
(DOCX)

S5 Table Association of treatment intensification with superior HbA1C control, with the level used in the next annual performance report. Abbreviations: BMI- body mass index; CCI- Charlson Comorbidity Index; OAD- oral antidiabetes agent; SD- standard deviation.
(DOCX)

S6 Table Association of treatment intensification with poor HbA1C control, with the levels used in the next annual performance report. Abbreviations: BMI- body mass index; CCI- Charlson Comorbidity Index; OAD- oral antidiabetes agent; SD- standard deviation.
(DOCX)
