# Supplementary material for: Assessment of the relationship between diabetes treatment intensification and quality measure performance using electronic medical records
Source: PLoS One. 2018 Jun 12;13(6):e0199011. doi: 10.1371/journal.pone.0199011 (PMC5997332; doi:10.1371/journal.pone.0199011)
Supplement: S1 Table — Abbreviations: BMI- body mass index; CCI- Charlson Comorbidity Index; OAD- oral antidiabetes agent; SD- standard deviation. (DOCX) [file pone.0199011.s001.docx]

Supplemental table 1: Patients’ treatment intensification and baseline characteristics by superior HbA1C control status, using the HbA1C level immediately after treatment intensification window

|  | **All Patients**  (N = 480) | **Poor control** | | |
| --- | --- | --- | --- | --- |
|  |  | **No**  (N = 240) | **Yes**  (N = 240) | **P-value** |
| **Treatment intensification** | | | | 0.0624 |
| Yes | 192 | 86 (44.79%) | 106 (55.21%) |  |
| No | 288 | 154 (53.47%) | 134 (46.53%) |  |
| **Index HbA1C result category*** | | | | <0.0001* |
| Moderate control | 285 | 108 (37.89%) | 177 (62.11%) |  |
| Superior control | 195 | 132 (67.69%) | 63 (32.31%) |  |
| **Age (years)*** | | | | 0.0213* |
| Mean (SD) | 58.9 (9.46) | 57.84 (9.7) | 59.96 (9.11) |  |
| **Sex** | | | | 0.7041 |
|  |  |  |  |  |
| Male | 306 | 151 (49.35%) | 155 (50.65%) |  |
| **Race/Ethnicity** | | | | 0.1113 |
| White | 328 | 167 (50.91%) | 161 (49.09%) |  |
| Hispanic | 36 | 24 (66.67%) | 12 (33.33%) |  |
| Black | 31 | 14 (45.16%) | 17 (54.84%) |  |
| Asian | 21 | 10 (47.62%) | 11 (52.38%) |  |
| Other/Unknown | 64 | 25 (39.06%) | 39 (60.94%) |  |
| **CCI** | | | | 0.9571 |
| Mean (SD) | 1.38 (0.92) | 1.38 (0.89) | 1.39 (0.94) |  |
| **CCI category** | | | | 0.7165 |
| 1 | 382 | 191 (50.00%) | 191 (50.00%) |  |
| 2 | 42 | 23 (54.76%) | 19 (45.24%) |  |
| 3+ | 56 | 26 (46.43%) | 30 (53.57%) |  |
| **BMI** | | | | 0.0873 |
| Mean (SD) | 32.95 (6.54) | 33.55 (6.69) | 32.35 (6.35) |  |
| **Insurance type** | | | | 0.376 |
| Commercial | 324 | 169 (52.16%) | 155 (47.84%) |  |
| Medicare | 153 | 69 (45.10%) | 84 (54.90%) |  |
| Other/Unknown | 3 | 2 (66.67%) | 1 (33.33%) |  |
| **Patient assigned provider specialty** | | | | 0.1724 |
| Endocrinology, Diabetes & Metabolism | 184 | 96 (52.17%) | 88 (47.83%) |  |
| Internal Medicine | 143 | 65 (45.45%) | 78 (54.55%) |  |
| Family Practice | 96 | 55 (57.29%) | 41 (42.71%) |  |
| All other specialties | 57 | 24 (42.11%) | 33 (57.89%) |  |
| **Number of OAD class used during baseline** | | | | 0.1823 |
| 1 | 213 | 95 (44.60%) | 118 (55.40%) |  |
| 2 | 173 | 95 (54.91%) | 78 (45.09%) |  |
| 3 | 75 | 41 (54.67%) | 34 (45.33%) |  |
| 4 | 19 | 9 (47.37%) | 10 (52.63%) |  |

* P<0.05

*Abbreviations: BMI- body mass index; CCI- Charlson Comorbidity Index; OAD- oral antidiabetes agent; SD- Standard deviation*
