# Supplementary material for: Assessment of the relationship between diabetes treatment intensification and quality measure performance using electronic medical records
Source: PLoS One. 2018 Jun 12;13(6):e0199011. doi: 10.1371/journal.pone.0199011 (PMC5997332; doi:10.1371/journal.pone.0199011)
Supplement: S2 Table — Abbreviations: BMI- body mass index; CCI- Charlson Comorbidity Index; OAD- oral antidiabetes agent; SD- standard deviation. (DOCX) [file pone.0199011.s002.docx]

Supplemental table 2: Patients’ treatment intensification and baseline characteristics by superior HbA1C control status, with the HbA1C level used in the next annual performance report

|  | **All Patients**  (N = 480) | **Superior HbA1C control** | | |
| --- | --- | --- | --- | --- |
|  |  | **No**  (N = 232) | **Yes**  (N = 248) | **P-value** |
| **Treatment intensification** | | | | 0.2049 |
| Yes | 192 | 86 (44.79%) | 106 (55.21%) |  |
| No | 288 | 146 (50.69%) | 142 (49.31%) |  |
| **Index HbA1C result category*** | | | | <0.0001* |
| Moderate control | 285 | 103 (36.14%) | 182 (63.86%) |  |
| Poor control | 195 | 129 (66.15%) | 66 (33.85%) |  |
| **Age (years)*** | | | | 0.0181* |
| Mean (SD) | 58.9 (9.46) | 57.81 (9.55) | 59.92 (9.27) |  |
| **Sex** | | | | 0.2623 |
|  |  |  |  |  |
| Male | 306 | 142 (46.41%) | 164 (53.69%) |  |
| **Race/Ethnicity** | | | | 0.0382* |
| White | 328 | 154 (46.95%) | 174 (53.05%) |  |
| Hispanic | 36 | 26 (72.22%) | 10 (27.78%) |  |
| Black | 31 | 16 (51.61%) | 15 (48.39%) |  |
| Asian | 21 | 10 (47.62%) | 11 (52.38%) |  |
| Other/Unknown | 64 | 26 (40.63%) | 38 (59.38%) |  |
| **CCI** | | | | 0.7038 |
| Mean (SD) | 1.38 (0.92) | 1.40 (0.93) | 1.36 (0.90) |  |
| **CCI category** | | | | 0.8591 |
| 1 | 382 | 183 (47.91%) | 199 (52.09%) |  |
| 2 | 42 | 22 (52.38%) | 20 (47.62%) |  |
| 3+ | 56 | 27 (48.21%) | 29 (51.79%) |  |
| **BMI** | | | | 0.1025 |
| Mean (SD) | 32.95 (6.54) | 33.50 (6.61) | 32.43 (6.45) |  |
| **Insurance type** | | | | 0.4444 |
| Commercial | 324 | 162 (50.00%) | 162 (50.00%) |  |
| Medicare | 153 | 68 (44.44%) | 85 (55.56%) |  |
| Other/Unknown | 3 | 2 (66.67%) | 1 (33.33%) |  |
| **Patient assigned provider specialty** | | | | 0.104 |
| Endocrinology, Diabetes & Metabolism | 184 | 92 (50.00%) | 92 (50.00%) |  |
| Internal Medicine | 143 | 62 (43.36%) | 81 (56.64%) |  |
| Family Practice | 96 | 55 (57.29%) | 41 (42.71%) |  |
| All other specialties | 57 | 23 (40.35%) | 34 (59.65%) |  |
| **Number of OAD class used during baseline** | | | | 0.4953 |
| 1 | 213 | 95 (44.60%) | 118 (55.40%) |  |
| 2 | 173 | 88 (50.87%) | 85 (49.13%) |  |
| 3 | 75 | 40 (53.33%) | 35 (46.67%) |  |
| 4 | 19 | 9 (47.37%) | 10 (52.63%) |  |

* P<0.05

*Abbreviations: BMI- body mass index; CCI- Charlson Comorbidity Index; OAD- oral antidiabetes agent; SD- Standard deviation*
