# Supplementary material for: Assessment of the relationship between diabetes treatment intensification and quality measure performance using electronic medical records
Source: PLoS One. 2018 Jun 12;13(6):e0199011. doi: 10.1371/journal.pone.0199011 (PMC5997332; doi:10.1371/journal.pone.0199011)
Supplement: S3 Table — Abbreviations: BMI- body mass index; CCI- Charlson Comorbidity Index; OAD- oral antidiabetes agent; SD- standard deviation. (DOCX) [file pone.0199011.s003.docx]

Supplemental table 3: Patients’ treatment intensification and baseline characteristics by poor HbA1C control status, using the HbA1C level immediately after treatment intensification window

|  | **All Patients**  (N = 480) | **Poor HbA1C control** | | |
| --- | --- | --- | --- | --- |
|  |  | **No**  (N = 351) | **Yes**  (N = 129) | **P-value** |
| **Treatment intensification** | | | | 0.8997 |
| Yes | 192 | 141 (73.44%) | 51 (26.56%) |  |
| No | 288 | 210 (72.92%) | 78 (27.08%) |  |
| **Index HbA1C result category*** | | | | <0.0001* |
| Moderate control | 285 | 244 (85.61%) | 41 (14.39%) |  |
| Poor control | 195 | 107 (54.87%) | 88 (45.13%) |  |
| **Age (years)*** | | | | 0.0109* |
| Mean (SD) | 58.9 (9.46) | 59.54 (9.31) | 57.16 (9.67) |  |
| **Sex** | | | | 0.262 |
|  |  |  |  |  |
| Male | 306 | 229 (74.84%) | 77 (25.16%) |  |
| **Race/Ethnicity** | | | | 0.0647 |
| White | 328 | 242 (73.78%) | 86 (26.22%) |  |
| Hispanic | 36 | 19 (52.78%) | 17 (47.22%) |  |
| Black | 31 | 24 (77.42%) | 7 (22.58%) |  |
| Asian | 21 | 16 (76.19%) | 5 (23.81%) |  |
| Other/Unknown | 64 | 50 (78.13%) | 14 (21.88%) |  |
| **CCI** | | | | 0.6903 |
| Mean (SD) | 1.38 (0.92) | 1.38 (0.94) | 1.39 (0.85) |  |
| **CCI category** | | | | 0.8219 |
| 1 | 382 | 281 (73.56%) | 101 (26.44%) |  |
| 2 | 42 | 29 (69.05%) | 13 (30.95%) |  |
| 3+ | 56 | 41 (73.21%) | 15 (26.79%) |  |
| **BMI** | | | | 0.1728 |
| Mean (SD) | 32.95 (6.54) | 32.67 (6.51) | 33.69 (6.61) |  |
| **Insurance type** | | | | 0.1678 |
| Commercial | 324 | 229 (70.68%) | 95 (29.32%) |  |
| Medicare | 153 | 119 (77.78%) | 34 (22.22%) |  |
| Other/Unknown | 3 | 3 (100%) |  |  |
| **Patient assigned provider specialty** | | | | 0.1372 |
| Endocrinology, Diabetes & Metabolism | 184 | 134 (72.83%) | 50 (27.17%) |  |
| Internal Medicine | 143 | 111 (77.62%) | 32 (22.38%) |  |
| Family Practice | 96 | 62 (64.58%) | 34 (35.42%) |  |
| All other specialties | 57 | 44 (77.19%) | 13 (22.81%) |  |
| **Number of OAD class used during baseline** | | | | 0.8039 |
| 1 | 213 | 159 (74.65%) | 54 (25.35%) |  |
| 2 | 173 | 123 (71.10%) | 50 (28.90%) |  |
| 3 | 75 | 54 (72.00%) | 21 (28.00%) |  |
| 4 | 19 | 15 (78.95%) | 4 (21.05%) |  |

*P<0.05

*Abbreviations: BMI- body mass index; CCI- Charlson Comorbidity Index; OAD- oral antidiabetes agent; SD- Standard deviation*
