# Supplementary material for: Assessment of the relationship between diabetes treatment intensification and quality measure performance using electronic medical records
Source: PLoS One. 2018 Jun 12;13(6):e0199011. doi: 10.1371/journal.pone.0199011 (PMC5997332; doi:10.1371/journal.pone.0199011)
Supplement: S4 Table — Abbreviations: BMI- body mass index; CCI- Charlson Comorbidity Index; OAD- oral antidiabetes agent; SD- standard deviation. (DOCX) [file pone.0199011.s004.docx]

Supplemental table 4: Patients’ treatment intensification and baseline characteristics by poor HbA1C control status, with the HbA1C level used in the next annual performance report

|  | **All Patients**  N = 480 | **Poor HbA1C control** | | |
| --- | --- | --- | --- | --- |
|  |  | **No**  (N = 370) | **Yes**  (N = 110) | **P-value** |
| **Treatment intensification** | | | | 1 |
| Yes | 192 | 148 (77.08%) | 44 (22.92%) |  |
| No | 288 | 222 (77.08%) | 66 (22.92%) |  |
| **Index HbA1C result category*** | | | | <0.0001* |
| Moderate control | 285 | 254 (89.12%) | 31 (10.88%) |  |
| Poor control | 195 | 116 (59.49%) | 79 (40.51%) |  |
| **Age (years)*** | | | | 0.0253* |
| Mean (SD) | 58.9 (9.46) | 59.43 (9.39) | 57.11 (9.50) |  |
| **Sex** | | | | 0.4802 |
|  |  |  |  |  |
| Male | 306 | 239 (78.10%) | 67 (21.90%) |  |
| **Race/Ethnicity** | | | | 0.0346* |
| White | 328 | 259 (78.96%) | 69 (21.04%) |  |
| Hispanic | 36 | 20 (55.56%) | 16 (44.44%) |  |
| Black | 31 | 25 (80.65%) | 6 (19.35%) |  |
| Asian | 21 | 16 (76.19%) | 5 (23.81%) |  |
| Other/Unknown | 64 | 50 (78.13%) | 14 (21.88%) |  |
| **CCI** | | | | 0.7477 |
| Mean (SD) | 1.38 (0.92) | 1.39 (0.94) | 1.35 (0.82) |  |
| **CCI category** | | | | 0.8227 |
| 1 | 382 | 293 (76.70%) | 89 (23.30%) |  |
| 2 | 42 | 34 (80.95%) | 8 (19.05%) |  |
| 3+ | 56 | 43 (76.79%) | 13 (23.21%) |  |
| **BMI** | | | | 0.3513 |
| Mean (SD) | 32.95 (6.54) | 32.78 (6.52) | 33.51 (6.61) |  |
| **Insurance type** | | | | 0.095 |
| Commercial | 324 | 241 (74.38%) | 83 (25.62%) |  |
| Medicare | 153 | 126 (82.35%) | 27 (17.65%) |  |
| Other/Unknown | 3 | 3 (100%) |  |  |
| **Patient assigned provider specialty** | | | | 0.0957 |
| Endocrinology, Diabetes & Metabolism | 184 | 144 (78.26%) | 40 (21.74%) |  |
| Internal Medicine | 143 | 116 (81.12%) | 27 (18.88%) |  |
| Family Practice | 96 | 65 (67.71%) | 31 (32.29%) |  |
| All other specialties | 57 | 45 (78.95%) | 12 (21.05%) |  |
| **Number of OAD class used during baseline** | | | | 0.8619 |
| 1 | 213 | 165 (77.46%) | 48 (22.54%) |  |
| 2 | 173 | 131 (75.72%) | 42 (24.28%) |  |
| 3 | 75 | 58 (77.33%) | 17 (22.67%) |  |
| 4 | 19 | 16 (84.21%) | 3 (15.79%) |  |

* P<0.05

*Abbreviations: BMI- body mass index; CCI- Charlson Comorbidity Index; OAD- oral antidiabetes agent; SD- Standard deviation*
