# Supplementary material for: Assessment of the relationship between diabetes treatment intensification and quality measure performance using electronic medical records
Source: PLoS One. 2018 Jun 12;13(6):e0199011. doi: 10.1371/journal.pone.0199011 (PMC5997332; doi:10.1371/journal.pone.0199011)
Supplement: S5 Table — Abbreviations: BMI- body mass index; CCI- Charlson Comorbidity Index; OAD- oral antidiabetes agent; SD- standard deviation. (DOCX) [file pone.0199011.s005.docx]

Supplemental table 5: Association of treatment intensification with superior HbA1C control, with the HbA1C level used in the next annual performance report

|  | **Estimate** | **Standard  Error** | **Odds Ratio** | **P-value** |
| --- | --- | --- | --- | --- |
| **Timely treatment intensification*** | 0.4993 | 0.2168 | 1.6476 | 0.0218* |
| **Physician characteristics** | | | | |
| Age | -0.0114 | 0.03612 | 0.9887 | 0.7525 |
| Male | -0.4726 | 0.3259 | 0.6234 | 0.1478 |
| **Physician specialty (compared to Family Medicine)** | | | | |
| Endocrinology, Diabetes & Metabolism | 0.1985 | 0.3043 | 1.2196 | 0.5146 |
| Internal Medicine | 0.5382 | 0.3198 | 1.7129 | 0.0931 |
| All other specialties | 0.6764 | 0.3938 | 1.9668 | 0.0866 |
| **Year in practice** | 0.01604 | 0.03677 | 1.0162 | 0.663 |
| **Average patient volume per month** | -0.00084 | 0.001402 | 0.9992 | 0.5496 |
| **Patient characteristics** | | | | |
| Age | 0.02068 | 0.0142 | 1.0209 | 0.1462 |
| Male * | 0.4837 | 0.2279 | 1.6221 | 0.0343* |
| **Race/Ethnicity (compared to White)** | | | | |
| Black | -0.3114 | 0.429 | 0.7324 | 0.4683 |
| Hispanic* | -1.2322 | 0.4712 | 0.2917 | 0.0093* |
| All others | 0.2216 | 0.2752 | 1.2481 | 0.4211 |
| **BMI** | -0.02627 | 0.01588 | 0.9741 | 0.0987 |
| **CCI category (compared to 1)** | | | | |
| 2 | -0.1728 | 0.3578 | 0.8413 | 0.6295 |
| 3+ | -0.1607 | 0.3243 | 0.8515 | 0.6205 |
| **Insurance type (compared to Commercial)** | | | | |
| Medicare & Other | -0.1299 | 0.2723 | 0.8782 | 0.6336 |
| **Index HbA1C results category (compared to Moderate control)** | | | | |
| Poor control* | -1.3714 | 0.2162 | 0.2538 | <.0001* |
| **Number of OAD types used during baseline (compared to 1)** | | | | |
| 2 | -0.2851 | 0.2351 | 0.7519 | 0.2259 |
| 3 | -0.3929 | 0.3042 | 0.6751 | 0.1973 |
| 4 | 0.000086 | 0.5369 | 1.0001 | 0.9999 |

* P<0.05

*Abbreviations: BMI- body mass index; CCI- Charlson Comorbidity Index; OAD- oral antidiabetes agent; SD- Standard deviation*
