# Supplementary material for: Assessment of the relationship between diabetes treatment intensification and quality measure performance using electronic medical records
Source: PLoS One. 2018 Jun 12;13(6):e0199011. doi: 10.1371/journal.pone.0199011 (PMC5997332; doi:10.1371/journal.pone.0199011)
Supplement: S6 Table — Abbreviations: BMI- body mass index; CCI- Charlson Comorbidity Index; OAD- oral antidiabetes agent; SD- standard deviation. (DOCX) [file pone.0199011.s006.docx]

Supplemental table 6: Association of treatment intensification with Poor HbA1C control, with the HbA1C level used in the next annual performance report

|  | **Estimate** | **Standard  Error** | **Odds Ratio** | **P-value** |
| --- | --- | --- | --- | --- |
| **Timely treatment intensification** | -0.3345 | 0.2575 | 0.7157 | 0.1947 |
| **Physician characteristics** | | | | |
| Age | -0.02285 | 0.04337 | 0.9774 | 0.5986 |
| Male | 0.5286 | 0.4069 | 1.6966 | 0.1946 |
| **Physician specialty (compared to Family Medicine)** | | | | |
| Endocrinology, Diabetes & Metabolism | -0.4599 | 0.3448 | 0.6313 | 0.183 |
| Internal Medicine* | -0.9205 | 0.3767 | 0.3983 | 0.0149* |
| All other specialties | -0.8356 | 0.4661 | 0.4336 | 0.0737 |
| **Year in practice** | 0.01901 | 0.04416 | 1.0192 | 0.6671 |
| **Average patient volume per month** | -0.00145 | 0.001644 | 0.9986 | 0.3781 |
| **Patient characteristics** | | | | |
| Age | -0.00258 | 0.01675 | 0.9974 | 0.8779 |
| Male* | -0.5485 | 0.2733 | 0.5778 | 0.0454* |
| **Race/Ethnicity (compared to White)** | | | | |
| Black | -0.05475 | 0.5464 | 0.9467 | 0.9202 |
| Hispanic* | 1.306 | 0.4886 | 3.6914 | 0.0078* |
| All others | 0.07205 | 0.3304 | 1.0747 | 0.8275 |
| **BMI** | 0.0203 | 0.01862 | 1.0205 | 0.2762 |
| **CCI category (compared to 1)** | | | | |
| 2 | -0.3448 | 0.4608 | 0.7084 | 0.4547 |
| 3+ | 0.08204 | 0.3902 | 1.0855 | 0.8336 |
| **Insurance type (compared to Commercial)** | | | | |
| Medicare & Other | -0.3573 | 0.3423 | 0.6996 | 0.2972 |
| **Index HbA1C results category (compared to Moderate control)** | | | | |
| Poor control* | 1.8406 | 0.2629 | 6.3003 | <.0001* |
| **Number of OAD types used during baseline (compared to 1)** | | | | |
| 2 | 0.1508 | 0.2805 | 1.1628 | 0.5911 |
| 3 | 0.1185 | 0.3702 | 1.1258 | 0.7492 |
| 4 | -0.4532 | 0.7233 | 0.6356 | 0.5313 |

* P<0.05

*Abbreviations: BMI- body mass index; CCI- Charlson Comorbidity Index; OAD- oral antidiabetes agent*
